# Human Influenza Surveillance: the Demand to Expand

**DOI:** 10.3201/eid1204.051198

**Published:** 2006-04

**Authors:** Scott P. Layne

**Affiliations:** * University of California at Los Angeles, Los Angeles, California, USA

**Keywords:** Influenza, surveillance, public health, vaccines, antivirals, high-throughput, laboratory methods, epidemiology, phenotype, genotype, perspective

## Abstract

The World Health Organization Influenza Program is one of the best developed and longest running infectious disease surveillance systems that exists. It maintains a worldwide watch of influenza's evolution to assist delivery of appropriately formulated vaccines in time to blunt seasonal epidemics and unpredictable pandemics. Despite the program's success, however, much more is possible with today's advanced technologies. This article summarizes ongoing human influenza surveillance activities worldwide. It shows that the technology to establish a high-throughput laboratory network that can process and test influenza viruses more quickly and more accurately is available. It also emphasizes the practical public health and scientific applications of such a network.

## Abstract 2

The potential of avian A/H5N1 to cause a global human pandemic is uncertain because it cannot be predicted with current knowledge.

Influenza strikes persons in developing and industrialized countries alike and is capable of killing healthy persons of all ages. Among the hardest hit are infants <1 year of age and adults >65. During any given year, influenza epidemics kill 500,000–1,000,000 persons globally, and an unpredictable pandemic is capable of killing millions (1). Yet death rate statistics alone do not capture the full impact of influenza; it causes many hospitalizations, secondary bacterial pneumonias, and middle ear infections in infants and young children (2). Worldwide, literally tons of antimicrobial drugs are used to treat these complications, and the economic consequences are enormous. For large populations, the only way to deter influenza is to administer vaccines targeted against ever-mutating strains (3).

## Current Surveillance

**Figure 1:**
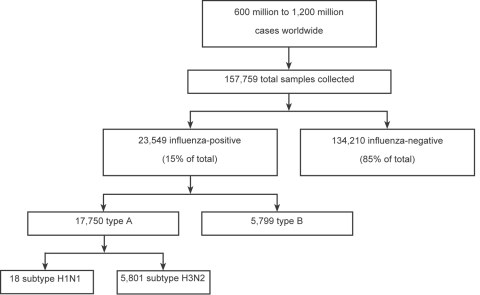
Global influenza surveillance for the 2004–2005 season Respiratory samples were collected from persons with febrile respiratory illness worldwide. Approximately 15% of samples were influenza positive. Note that only some of the type A viruses were subtyped. Data are from the Centers for Disease Control and Prevention website (http://www.cdc.gov/flu/weekly/weeklyarchives2004-2005/04-05summary.htm).

The World Health Organization (WHO) Influenza Program was established in 1952 to assist with public health threats associated with influenza. Today, its network of 112 national centers in 83 countries collects ≈160,000 samples each year from 600 to 1,200 million persons with influenza. As shown in Figure 1, the centers screen samples for influenza viruses and type- (A versus B) and subtype- (e.g., A/H1N1, A/H3N2) relevant samples (4). Certain influenza-positive samples are then forwarded to 1 of 4 WHO collaborating centers for further immunologic and genetic characterizations. Twice a year, WHO organizes a formal meeting with its collaborating center directors to review information on circulating influenza strains. This advisory committee identifies circulating strains that new vaccine formulations should target. Because influenza epidemics peak during the winter months, the committee offers its recommendations in February and September for the Northern and Southern Hemispheres, respectively. The findings are then reviewed by national health authorities who approve, and occasionally amend, implementation of the recommended vaccine strains (5). Surveillance for influenza requires global and national monitoring for both virus and disease activity to determine when, where, and which influenza viruses are circulating in the United States and globally, to determine the intensity and impact of influenza activity on defined health outcomes and identify unusual or severe outbreak, and to detect the emergence of novel influenza viruses that may cause a pandemic.

The Centers for Disease Control and Prevention's (CDC) WHO Collaborating Center for Reference and Research on Influenza supplies standardized reagents and test kits to all national centers for detecting influenza A and B strains, subtyping strains, and determining whether sample strains are immunologically related to recent vaccine strains. Typical circulating strains and atypical ones that appear to differ from vaccine strains are forwarded to 1 of 4 collaborating centers (in the United States, United Kingdom, Australia, and Japan) for further characterization (5). During the 2004–2005 influenza season, CDC's center in Atlanta received ≈3,500 strains from domestic and foreign surveillance. A partial summary of laboratory methods used is outlined below.

In most situations, 6–10 serum samples are used to compare sample strains against vaccine and reference strains, and during any given influenza season, >99% of sample strains are successfully matched by routine hemagglutination inhibition (HI) assays. Laboratory workers replicate sample strains in cell cultures or fertile eggs and adjust the resulting viral stocks to a standard hemagglutinin titer. They then use HI assays to determine whether sample strains are immunologically related (i.e., cross-reactive) to recent vaccine strains. Typing sera are added to wells in a series of 2-fold dilutions, and after the reactions stabilize, laboratory workers score assay wells (positive or negative) by looking for agglutinated erythrocytes (positive) that do not form buttons at the bottom of plates versus non-agglutinated cells (negative) that do form buttons. For sample strains that behave as variants, laboratory workers inject them into ferrets to produce a strain-specific antiserum. When the new antiserum is ready, HI assays are again performed as above. If the new sera show significant gaps in cross-reactivity (usually defined as a 4-fold difference between sample and vaccine strains), they are incorporated into the routine laboratory set and used to look for new epidemic strains. Examples of strains that were identified as variants include A/H3N2/Sydney, which spread rapidly among persons in 1997, and A/H5N1/Hong Kong, which jumped from fowl to humans in 1997. During the 2004–2005 influenza season, ≈3,500 HI assays were performed.

During the 2004–2005 influenza season, >600 cleaved hemagglutinin (HA1) domain sequences were analyzed. In this process, laboratory workers extract viral RNA from samples, convert viral RNA to cDNA, amplify cDNA with polymerase chain reaction (PCR) primers, and analyze DNA products with capillary array sequencers. They then compare sample strain sequences against vaccine and reference strain sequences to determine their phylogenetic relationships.

During the 2004–2005 influenza season, ≈350 neuraminidase (NA) sequences were analyzed to determine their phylogenetic relationships. For this process, laboratory workers follow similar preparative steps used for HA1 and then use multiple PCR primers to sequence the entire gene segment.

During the 2004–2005 influenza season, ≈3,500 single nucleotide polymorphism (SNP) profiles were analyzed from circulating strains. For this analysis, laboratory workers follow similar preparative steps used for HA1 and then use pyrosequencing to detect SNP that confer resistance to the antiviral drugs amantadine and rimantadine.

During the 2004–2005 influenza season, ≈120 whole genomes were sequenced from circulating strains. In this process, laboratory workers extract viral RNA from samples, convert viral RNA to cDNA, amplify cDNA with PCR primers, and analyze DNA products with capillary array sequencers. Amplification of all 8 gene segments (PB1, PB2, PA, HA, NP, NA, M1/M2, NS1/NEP) that have a combined length of ≈13.6 kb requires ≈30 type- and subtype-specific PCR primers.

These activities give ≈6 months to vaccine manufacturers on either side of the equator for scale up, production, and distribution. These activities also give healthcare services another 3 months to administer the ≈250 million doses of trivalent vaccine that are used globally. Despite its sophistication and scale, however, the WHO Influenza Program has several shortcomings (6).

First, surveillance gaps exist in many parts of the world for a variety of reasons, including limited funding, lack of infrastructure support for surveillance teams and preserving influenza samples, and intentional underreporting at the national level (7). Second, current laboratory methods for characterizing influenza are time-consuming and labor-intensive and, as a result, relatively few viral strains undergo definitive phenotyping and genotyping assays (5,8,9). During the 2004–2005 influenza season, for example, the 4 WHO collaborating centers analyzed ≈6,000 strains, representing only 1 sample per 100,000 influenza cases worldwide (Figure 1). Far fewer strains from domestic poultry and swine or from wild aquatic birds, which are thought to serve as precursors for pandemic strains, are analyzed comprehensively in a given year (10).

Third, with current methods, it can take weeks to months to generate laboratory data on influenza samples and understand their significance (Appendix). Such prolonged times can impede vaccine strain selection activities. For example, the vaccine administered throughout North America in 1997 provided inadequate protection against the A/H3N2 Sidney strain that spread rapidly from Asia and Australia (11). Public health officials ascribed the poor match between circulating strains and vaccine strains to many factors including 1) less than optimal surveillance, 2) time required to prepare and ship isolates, 3) lag time in laboratory testing with current manual methods, and 4) rapid spread of the Sydney variant.

Although the program has a remarkably good track record, it did not detect the Sydney variant in time to include it in the vaccine before the epidemic. Failure to detect an emerging influenza virus could prove disastrous should it be a novel strain with pandemic potential (12). The 1918 influenza pandemic is the biggest infectious disease catastrophe on record, topping even the medieval Black Death. Within months after the initial outbreak, the A/H1N1 virus struck 500 million persons and killed 40–50 million worldwide when the total population was only 2 billion (13). Subsequent pandemics, brought about by a shift to A/H2N2 in 1957 and A/H3N2 in 1968, had far lower death rates.

Between March and May of 1997, an outbreak of avian A/H5N1 in Hong Kong killed a child who was otherwise healthy and thousands of chickens (14). For the next 6 months, no new cases appeared. Between November and December of 1997, avian A/H5N1 infected 17 people and killed 5 of them (15,16). Confronted with a case-fatality ratio of 33%, health authorities took quick action and opted to destroy all 1.5 million chickens in Hong Kong. It took nearly 6 months to identify the index case; events transpired so quickly that manual laboratory methods were unable to generate all of the information that was needed.

Since 2004, avian A/H5N1 has caused additional outbreaks throughout Asia, resulting in >60 human deaths in Vietnam, Thailand, Cambodia, and Indonesia and the destruction of 150 million birds (17). In 2005, through a combination of wild bird migrations and farming practices, highly pathogenic avian influenza spread to northern China, Mongolia, Tibet, Kazakhstan, and Russia (18). At the time of this writing, it had further spread to several European countries (Turkey, Romania, and Greece) and threatened to spread to other continents, including Africa and North America through avian flyways.

The potential of avian A/H5N1 to cause a global human pandemic is uncertain because it cannot be predicted with current knowledge (19). Nevertheless, the anticipated economic, social, and political consequences are enormous (20,21). Therefore, we face a compelling demand to expand the current influenza surveillance system (6,22).

## High-throughput Network

**Figure 2:**
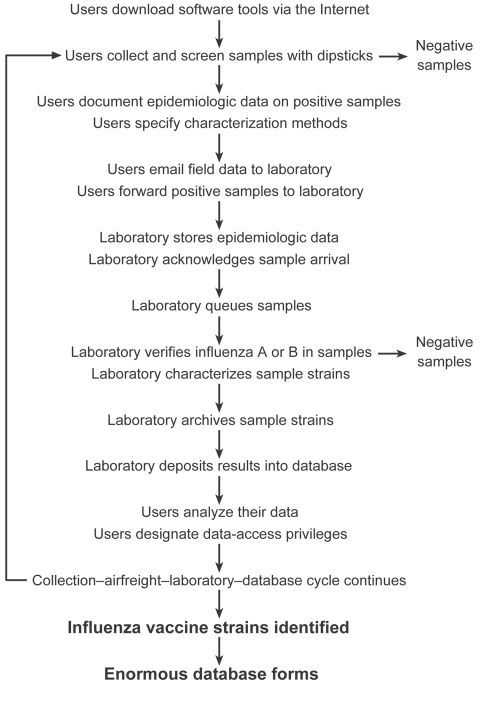
Flow chart for utilizing the high-throughput laboratory network.

In August 2004, the US Department of Health and Human Services released a draft of its Pandemic Influenza Response and Preparedness Plan. The plan's surveillance annex offered specific recommendations for system enhancements and next steps (5). Many of these enhancements could be achieved by developing a high-throughput laboratory network that would expand the capabilities of the existing WHO collaborating centers on influenza (6,22). With such enhancements, WHO national centers would be able to collect samples from people with febrile respiratory illnesses, record epidemiologic observations, and send samples directly to the high-throughput network. At each site, high-throughput automated systems would collaborate, and epidemiologic observations and test results would appear in the laboratory's web-enabled database for analysis within days (23). Internet-based capabilities would allow WHO national centers to examine their own data and improve surveillance in an iterative manner (Figure 2). In tracking changes in epidemic strains, the new system would facilitate nonbiased proportional sampling of persons with febrile respiratory illnesses in an iterative fashion. In detecting the emergence of novel strains with pandemic potential, the new system would facilitate the use of rapid and more sensitive methods.

The plan integrates available biologic, engineering, and informatic technologies into a networked capability and makes them available through the Internet (23). Influenza is well suited to this approach because of its obvious public health implications, but also because a well-established infrastructure that includes global surveillance, standardized laboratory methods, surveillance-based recommendations, and targeted vaccines already exists (6,22). Key elements are shown in Figure 2.

Platform-independent software that facilitates influenza surveillance would be provided by the high-throughput network (23). Internet-based tools would manage laboratory access, epidemiologic questionnaires, testing instructions, sample submission, data analysis, and data privileges. The release of data to other users or entities would be controlled by the submitting organization or authority. More importantly, the WHO committee and national health authorities that recommend and review vaccine strains and antiviral drugs would have access to all the data.

On average, 1 of every 6 samples collected from persons with febrile respiratory illnesses contains influenza A or B viruses (24). The remainder contain other viral and bacterial pathogens. To expedite data collection, surveillance teams could use influenza dipsticks to screen samples on the spot. Several companies make diagnostic kits for influenza A and B; although these tests have certain disadvantages (limited sensitivity and specificity of immunoassays), their underlying technologies can form the basis for improved influenza screening and sampling devices (2). Such influenza dipsticks—or even portable PCR-based assays—would make it easier for teams in the field to screen out negative samples and focus on documenting epidemiologic information on positive ones.

An epidemiologic questionnaire would be provided to surveillance teams, with a menu that covers key questions (23). What are the collection date and location? Who is the host (human versus animal)? What is the age of the host? What is the physiologic source of the sample? What is the observed or reported severity of illness? What is the observed or reported outcome of illness? Which influenza vaccine or antiviral agents were administered? What are the likely exposures? Is there any recent travel history? The questionnaire would run on inexpensive handheld devices (e.g., personal digital assistants) or cell phones. Bar codes would be used to link samples to their corresponding questionnaires. Completed questionnaires would be sent by email to the high-throughput laboratory network, where questionnaire and laboratory data would form the basis for seeking associations on factors that influence virulence, transmissibility, and host range (19).

Current high-throughput automated laboratory systems are capable of operating 24 hours a day. At each networked site, epidemiologic questionnaires and instructions would arrive by the Internet, and bar coded samples would arrive by air freight. Larger sites could operate systems for genotyping, phenotyping, replicating, and archiving influenza viruses. Smaller sites could operate systems for genotyping and archiving viruses. In serving as resources, each site would provide reagents and supplies for analyzing all influenza subtypes. They would also perform control assays on a daily basis and maintain a quality assurance program, the documentation of which would be stored in the database. Automated laboratory methods would build upon manual methods currently in use and, because they can reduce working (liquid) volumes by at least 5- to 10-fold, they would enable economies of scale (23,25).

Genotyping systems would have flexibility to sequence all 8 RNA segments or any individual segment from influenza viruses. The various steps performed would include transcription viral RNA into cDNA, selection of optimal PCR primers, amplification of DNA by PCR, and analysis by capillary array DNA sequencers (26–28). Influenza viruses are often propagated in cell cultures or embryonated eggs to obtain enough viral RNA for sequencing. Newer methods that avoid this growth step, however, would facilitate direct high-throughput analysis of native samples from surveillance, including active samples preserved by cold chain as well as inactive samples preserved by ethanol fixation (29).

Phenotyping systems would conduct HI and neuraminidase inhibition (NI) assays. HI assays are easily adaptable to automation, but they require relatively large quantities of virus and typing sera (8). To overcome this drawback, automated methods that use flow cytometry are under development (30). They work by attaching monoclonal or polyclonal typing sera to a set of color-coded (multiplexed) beads and detecting the interaction of influenza with such beads. A high-throughput system that performs HI assays in parallel with flow cytometer–based assays, for example, may offer the best means to test and validate improved influenza serotyping methods. NI assays are also easily adaptable to automation, particularly newer ones that use a chemiluminescent sialic acid substrate instead of a fluorogenic substrate (31). They work by mixing substrate with neuraminidase from sample strains and measuring the chemiluminescent signal over time. When performed over a range of inhibitor or antiviral drug (oseltamivir and zanamivir) concentrations, they enable the determination of the 50% inhibitory concentration (IC_50_) for individual drugs and strains (32).

A replicating system would verify that influenza A and B antigens are present in samples and set aside negative ones (33). Positive samples would be injected into cell cultures or embryonated eggs and, several days later, automatically harvested, assayed for HA titers, and adjusted to uniform concentrations. A part of this fresh stock would then go to the long-term storage system.

Archiving systems would store influenza samples for an extended time. The archiving system would take stocks from the replication system and place them into modular bar-coded storage containers, which would then be placed into freezers. Every step in the storage and retrieval process would be recorded by bar code scanners and managed by an inventory tracking program (23).

## Expanded Surveillance

Influenza virus evolves through a combination of point mutations (drifts) and reassortment events (shifts) in its gene segments. For vaccine strain selection, laboratory methods for characterizing influenza have focused primarily on changes in hemagglutinin and neuraminidase (and to a much lesser extent on the M2 ion channel protein) because immunity against these surface proteins is protective (2). The emphasis on immune-inducing proteins is clearly practical, but it may overlook changes in the remaining gene segments (26).

Whole-genome sequencing and phylogenetic analysis of 156 A/H3N2 viruses that infected humans in New York from 1999 to 2004 shows 2 substantial findings (28). The first is that multiple influenza strains co-circulated in humans over time, with strains falling into 1 of 3 distinct clades. The second is that mixing between these clades occurred over short intervals of time, resulting in at least 4 reassortment events among the strains analyzed (28). One such reassortment event (shift), rather than a point mutation (drift), appears to explain the emergence of the A/Fujian/411/2002-like strain that caused an epidemic during the 2003–2004 influenza season. Similar findings on A/H3N2 viruses that infected humans in Australia and New Zealand from 2003 to 2004 have also been reported (27). In this instance, swaps of neuraminidase and 3 internal genes (NS1/NEP, NP, M1/M2) were found. Both independent findings show that influenza A virus is less restricted than previously believed and that reassortment events can occur without warning. Altogether, these new findings underscore the importance of rapid, whole-genome analysis for future influenza surveillance (28).

The high-throughput laboratory network would give rise to 3 domains of associated data from surveillance (34). Epidemiologic data would pertain to dates, locations, hosts, outcomes, histories, and exposures. Genotypic data would pertain to the exact sequence of nucleotides in all 8 viral RNA segments. Phenotypic date would pertain to immunologic pedigrees (HI titers) and antiviral drug sensitivities (IC_50_) of sample strains. Some practical public health and scientific uses of such organized data follow.

New influenza vaccines are often introduced after 3 criteria have been met (25). First, a new strain is identified by laboratory-based methods. Second, geographic spread of the new strain is associated with human illness. Third, the most recent influenza vaccine stimulates a reduced immunologic response to the new strain. Given these criteria, the high-throughput laboratory network would help in 2 ways. It would provide faster information for vaccine strain selection, potentially saving 1–2 months in vaccine delivery. It would also continuously monitor for the emergence of escaping influenza strains and guide critical decisions to update pandemic vaccines or use them in combination with limited supplies of antiviral drugs (19). Researchers and drug companies are developing modern methods (based on reverse genetics and cell cultures, for example) to manufacture influenza vaccines that could cut delivery times in half (2,35). Within the next few years, these new methods, in combination with a high-throughput network, could save additional vaccine delivery time and save lives (6,19).

Fifteen hemagglutinin (H1–H15) and 9 neuraminidase (N1–N9) subtypes diverge by as much as 50% in their overall amino acid composition. Within each subtype, smaller amino acid substitutions (drifts) that enable influenza viruses to evade preexisting immunity exist (2,3). Although sequencing influenza viruses is useful for understanding viral mixing and evolution, it cannot delineate how immunologic (i.e., drift and shift) variants relate to one another at the amino acid and RNA coding levels. To develop such understanding, a large base of phenotypic data must be associated with its corresponding genotypic data. For each receptor subtype, phenotypic data would consist of HI titers and genotypic data would consist of RNA sequences from the same virus. Building a rough association matrix would be the first step in understanding how variants relate to one another at the amino acid and RNA levels.

Subsequently, a more complete association matrix would be used to develop models that could predict whether viral strains are immunologically related from sequences alone. Such efforts could help develop influenza vaccines that protect against a wider range of variants and establish a more fundamental molecular basis for influenza surveillance (25).

Researchers have proposed using antiviral drugs such as oseltamivir to halt an avian influenza outbreak in humans (36,37). The strategy would require stockpiling millions of doses and administering them to persons in the epicenter and surrounding areas within weeks. Immediate recognition of the outbreak and rapid surveillance to determine its size would be essential. Drug-resistant avian influenza viruses would likely emerge at some point, representing a potential threat to emergency control efforts, and health authorities would need real-time information on where the viruses were found and how many of them existed (38). Such emergency interventions would generate thousands of samples for laboratory analysis within days. Given current laboratory surge capacity, a high-throughput laboratory network may be the only feasible means to meet the challenge.

## Implementation

The spreading avian A/H5N1 outbreak poses serious threats to the health, economy, and security of the world (22). It has motivated political leaders and health officials to increase financial support for influenza surveillance and to seek agreements and incentives that promote information sharing and international cooperation (39). Effective measures will require real-time, accurate, and comprehensive information to make rapid public health decisions.

The first 2 sites in a high-throughput laboratory network could be up and running in 12–18 months at a cost of $15 million. It would generate epidemiologic, genotypic, and phenotypic data as described in this article. With available technologies and methods, each site would be capable of analyzing up to 10,000 samples per year, a substantial improvement over current capabilities. Implementing multiple sites worldwide (at the 4 WHO collaborating centers, for example) would enable regional collaborations and help encourage the timely sharing of samples and information (40).

Human history shows that an influenza pandemic is years overdue (41). Moreover, whole-genome sequencing of influenza virus shows dynamic RNA segments that are capable of epidemiologically meaningful reassortment events (27,28). Whether avian A/H5N1 will be the precursor strain is unknown. However, we must expand, speed up, and connect human and animal surveillance efforts today, which must be matched with an expanded capacity to produce and deliver influenza vaccines worldwide.

## Appendix

CDC's WHO Collaborating Center for Reference and Research on Influenza supplies standardized reagents and test kits to all national centers for detecting influenza A and B strains, subtyping strains, and determining whether sample strains are immunologically related to recent vaccine strains. Typical circulating strains and atypical ones that appear to differ from vaccine strains are forwarded to 1 of 4 collaborating centers (in the United States, United Kingdom, Australia, and Japan) for further characterization (5). During the 2004–2005 influenza season, CDC's center in Atlanta received ≈3,500 strains from domestic and foreign surveillance. A partial summary of laboratory methods used is outlined below.

In most situations, 6–10 serum samples are used to compare sample strains against vaccine and reference strains, and during any given influenza season, >99% of sample strains are successfully matched by routine hemagglutination inhibition (HI) assays. Laboratory workers replicate sample strains in cell cultures or fertile eggs and adjust the resulting viral stocks to a standard hemagglutinin titer. They then use HI assays to determine whether sample strains are immunologically related (i.e., cross-reactive) to recent vaccine strains. Typing sera are added to wells in a series of 2-fold dilutions, and after the reactions stabilize, laboratory workers score assay wells (positive or negative) by looking for agglutinated erythrocytes (positive) that do not form buttons at the bottom of plates versus non-agglutinated cells (negative) that do form buttons (8). For sample strains that behave as variants, laboratory workers inject them into ferrets to produce a strain-specific antiserum. When the new antiserum is ready, HI assays are again performed as above. If the new sera show significant gaps in cross-reactivity (usually defined as a 4-fold difference between sample and vaccine strains), they are incorporated into the routine laboratory set and used to look for new epidemic strains. Examples of strains that were identified as variants include A/H3N2/Sydney, which spread rapidly among persons in 1997, and A/H5N1/Hong Kong, which jumped from fowl to humans in 1997. During the 2004–2005 influenza season, ≈3,500 HI assays were performed.

During the 2004–2005 influenza season, >600 cleaved hemagglutinin (HA1) domain sequences were analyzed. In this process, laboratory workers extract viral RNA from samples, convert viral RNA to cDNA, amplify cDNA with PCR primers, and analyze DNA products with capillary array sequencers. They then compare sample strain sequences against vaccine and reference strain sequences to determine their phylogenetic relationships. During the 2004–2005 influenza season, ≈350 neuraminidase (NA) sequences were analyzed to determine their phylogenetic relationships. For this process, laboratory workers follow similar preparative steps used for HA1 and then use multiple polymerase chain reaction (PCR) primers to sequence the entire gene segment.

During the 2004–2005 influenza season, ≈3,500 single nucleotide polymorphism (SNP) profiles were analyzed from circulating strains. For this analysis, laboratory workers follow similar preparative steps used for HA1 and then use pyrosequencing to detect SNP that confer resistance to the antiviral drugs amantadine and rimantadine (9).

During the 2004–2005 influenza season, ≈120 whole genomes were sequenced from circulating strains. In this process, laboratory workers extract viral RNA from samples, convert viral RNA to cDNA, amplify cDNA with PCR primers, and analyze DNA products with capillary array sequencers. Amplification of all 8 gene segments (PB1, PB2, PA, HA, NP, NA, M1/M2, NS1/NEP) that have a combined length of ≈13.6 kb requires ≈30 type- and subtype-specific PCR primers.
